# Thrombotic activation before and after total hip arthroplasty. A prospective cohort study

**DOI:** 10.1186/s12891-021-04566-1

**Published:** 2021-08-13

**Authors:** Marta Burbul, Dariusz Tomaszewski, Anna Rogalska, Krzysztof Gawroński, Sławomir Literacki, Marcin Waśko

**Affiliations:** 1 Department of Traumatology and Orthopaedics, Military Institute of Medicine, Warsaw, Poland; 2 Department of Anesthesiology and Intensive Therapy, Military Institute of Medicine, Warsaw, Poland; 3 Department of Health Economics and Health Management, School of Health Sciences in Bytom, Medical University of Silesia in Katowice, Bytom, Poland; 4 Institute of Hematology and Transfusion Medicine, Warsaw, Poland; 5 Department of Laboratory Diagnostics, Military Institute of Medicine, Warsaw, Poland; 6 Department of Radiology and Imaging, The Medical Center of Postgraduate Education, Warsaw, Poland

**Keywords:** Blood loss, Total hip arthroplasty, Coagulation factors, Venous thromboembolism

## Abstract

**Background:**

Total hip arthroplasty (THA) causes acute blood loss. It may lead to a deficiency in coagulation factors, which, in turn, may lead to increased bleeding during the postoperative period.

**Methods:**

Thirty patients (18 women) with a mean age of 67 years (range: 63–72 years) participated in this prospective diagnostic study. THA was performed without tranexamic acid administration in the perioperative period. Activities of clotting factors II, VIII, X, and fibrinogen concentration were evaluated before surgery, 6 hours after the procedure, 2, 4, and 6 days after the operation. All laboratory tests were performed using ACL TOP 500 CTS analyzer.

**Results:**

No thromboembolic complications were noted during hospitalization. Mean fibrinogen concentration was 366 mg/dL before surgery, which decreased to 311 mg/dL 6 hours after the operation and peaked at 827 mg/dL on the 4th day after the procedure. Activities of factors II and X decreased on the second and fourth days after surgery. Although the activity of factor VIII decreased after the procedure, it remained within the normal range. Increased baseline fibrinogen concentrations were observed in 6 out of 30 (20%) patients. Mean blood loss was 1332 mL (range, 183–2479 mL) and did not correlate with changes in clotting factor activities.

**Conclusions:**

In patients undergoing THA, fibrinogen acts as an acute-phase protein. Activities of clotting factors II and X normalize within 6 days, and although the activity of factor VIII decreases, it remains within the normal range.

**Trial registration:**

The study was pre-registered May 1st, 2020 on ClinicalTrials.gov

## Background

Total hip arthroplasty (THA) leads to increased coagulation through ischemia, reduced perfusion, and inflammatory reaction [2]. Factors causing ischemia include, among others, loss of blood inherent to orthopedic procedures [28]. Acute blood loss decreases plasma fibrinogen concentration and activity of other clotting cascade proteins, which may aggravate further blood loss and delay hemostasis [20]. In patients undergoing cardiac, vascular, and spinal surgery, a decrease in plasma fibrinogen concentration is a critical risk factor for blood loss and the need for transfusions [3, 13, 18, 25, 33]. Transient deficiency of other coagulation factors may also increase bleeding during the perioperative period [5].

Various concepts have been proposed to reduce both blood loss in the surgical field and the volume of blood products transfused after the surgery. One such method involved prophylactic fibrinogen concentrate transfusion in the perioperative period [20]; however, increased fibrinogen concentration is associated with increased risk of thromboembolic complications, such as pulmonary embolism [15, 27, 31].

Strategies for preventing blood loss also include the blood salvage systems, postoperative flexion of the knee joint, hypotensive anesthesia, and tourniquet; however, the most widespread method for preventing blood loss is the use of anti-fibrinolytic agents, such as tranexamic acid (TXA), and ε-aminocaproic acid [16, 23]. Tranexamic acid can effectively reduce blood loss and transfusion rates in TKA without increasing the risk of deep vein thrombosis and pulmonary embolism [26, 35].

A deeper understanding of hemostasis after surgically induced trauma might help to devise better ways of identifying patients requiring optimization of the coagulation and fibrinolysis processes and managing blood loss after those procedures. Therefore, this study aimed to analyze changes in clotting factors II, VIII, and X and fibrinogen concentration in patients undergoing THA for idiopathic osteoarthritis during the first 6 days after surgery. We hypothesized that (1) blood loss correlates with diminished activities of clotting factors, and (2) fibrinogen concentrations will decrease immediately after THA and return to normal levels after that.

## Patients and methods

### Study design and setting

The local Bioethics Commission (Military Institute of Medicine) approved the study (84/WIM/2017) on December 20th, 2017). The study was performed accordingly with the tenets of the Declaration of Helsinki. The study was registered in the clinical trials registry (NCT04372173) on May 1st, 2020. Participation in the study was offered to all patients hospitalized in the Orthopedic Ward, who were eligible for hip arthroplasty for idiopathic osteoarthritis in the study period (from February to April 2018). Exclusion criteria were (1) hip osteoarthritis of non-idiopathic etiology, (2) history of venous thromboembolism (VTE) or arterial embolism, (3) anticoagulation treatment, (4) coagulopathy, (5) infection, and (6) renal failure defined as a creatinine clearance below 50 ml/min. Ultrasound examination was performed only in patients who had a clinical suspicion of VTE, and this decision was left to the discretion of the attending surgeon. All study participants gave written informed consent to participate in the study.

### Blood draws

Blood samples were collected as part of routine hemostatic parameter monitoring to determine clotting system parameters (before surgery); blood samples were also collected to determine blood cell counts (6 h post-surgery and fourth-day post-surgery) and, for the study alone, on the 2nd and sixth days post-surgery. Venous blood samples were collected from peripheral veins using 2.7 Ml BD Vacutainer tubes with 3.2% sodium citrate (Becton Dickinson, Franklin Lakes, NJ, USA). Other than the six-hour post-surgery data point, samples were collected at fixed hours (8 a.m.).

### Laboratory analysis

All clotting parameters (fibrinogen, factor II, factor VIII, factor X) were determined using an ACL TOP 500 CTS automated coagulation analyzer (Instrumentation Laboratories, Bedford, MA, USA).

Fibrinogen concentrations were determined by the Clauss coagulation method using HemosIL Q.F.A. Thrombin reagent (Bovine) (Instrumentation Laboratories, Bedford, MA, USA). The Clauss method measures the coagulation time of a patient’s diluted plasma after the addition of high concentrations of thrombin. Coagulation time is inversely proportional to fibrinogen concentration in the tested sample.

Clotting activities of factors II and X were measured by the coagulation method using HemosIL RecombiPlasTin 2G reagent (Instrumentation Laboratories, Bedford, MA, USA) and lyophilized substrate plasma: HemosIL Factor II deficient plasma (Instrumentation Laboratories, Bedford, MA, USA) and HemosIL Factor X deficient plasma (Instrumentation Laboratories, Bedford, MA, USA) to determine prothrombin time values. The patient’s plasma is diluted and added to plasma without a given coagulation factor (substrate plasma). Substrate plasma clotting time changes proportionally to the clotting factor activity in the patient sample.

Factor VIII activity was measured by the coagulation method using HemosIL SynthASil reagent (Instrumentation Laboratories, Bedford, MA, USA) and HemosIL Factor VIII deficient plasma lyophilized substrate plasma (Instrumentation Laboratories, Bedford, MA, USA) to determine the activated partial thromboplastin time. The measurement consists of adding diluted patient plasma to the substrate plasma and determining the activated partial thromboplastin time. Substrate plasma clotting time changes proportionally to the clotting factor activity in the patient sample.

### Blood loss calculation

We calculated blood loss using Mercuriali’s formula [17]. This formula is based on the preoperative hematocrit and fifth post-operative day hematocrit. The formula requires the patient’s blood volume calculated using the Nadler formula [19]. After calculation, we converted the results to milliliters of blood, based on the patient’s average hematocrit.

### Surgery

All patients were operated on by a single surgical team using a posterolateral approach, with cementless hip replacements implanted, and without tranexamic acid administered in the perioperative period. All patients underwent VTE prophylaxis (enoxaparin 40 mg 1 × 1 subcutaneously from the surgery day), per local guidelines [4].

### Demographics, description of the study population

The following data were entered into the database: age, body mass index (BMI), gender, perioperative risk assessment scale according to the American Society of Anesthesiologists score (ASA score), blood transfusions, hemoglobin levels, and hematocrit before surgery and 2 days post-surgery, and instances of thromboembolic complications. This study was observational and did not require any changes in the treatment of patients.

### Statistical analysis

Statistical analysis was performed using MedCalc 18.5 software (MedCalc, Ostend, Belgium), and normal distribution was verified using the Kolmogorov-Smirnov test. Continuous variables of normal distribution were presented as an average with a 95% confidence interval, minimum, and maximum values. In the absence of normal distribution, variables were transformed logarithmically, and their normality was retested. Student’s t-test for related variables was used to compare averages after logarithmic transformation. In the absence of a normal distribution of post logarithmic transformation, variables were compared using the Wilcoxon test. Medians were compared using the Mann-Whitney test. We used stepwise multiple regression to examine the correlation between blood loss and demographic parameters, concentrations, and changes in clotting factors. For hypothesis testing, values of *p* < 0.05 were considered statistically significant.

## Results

**Fig. 1:**
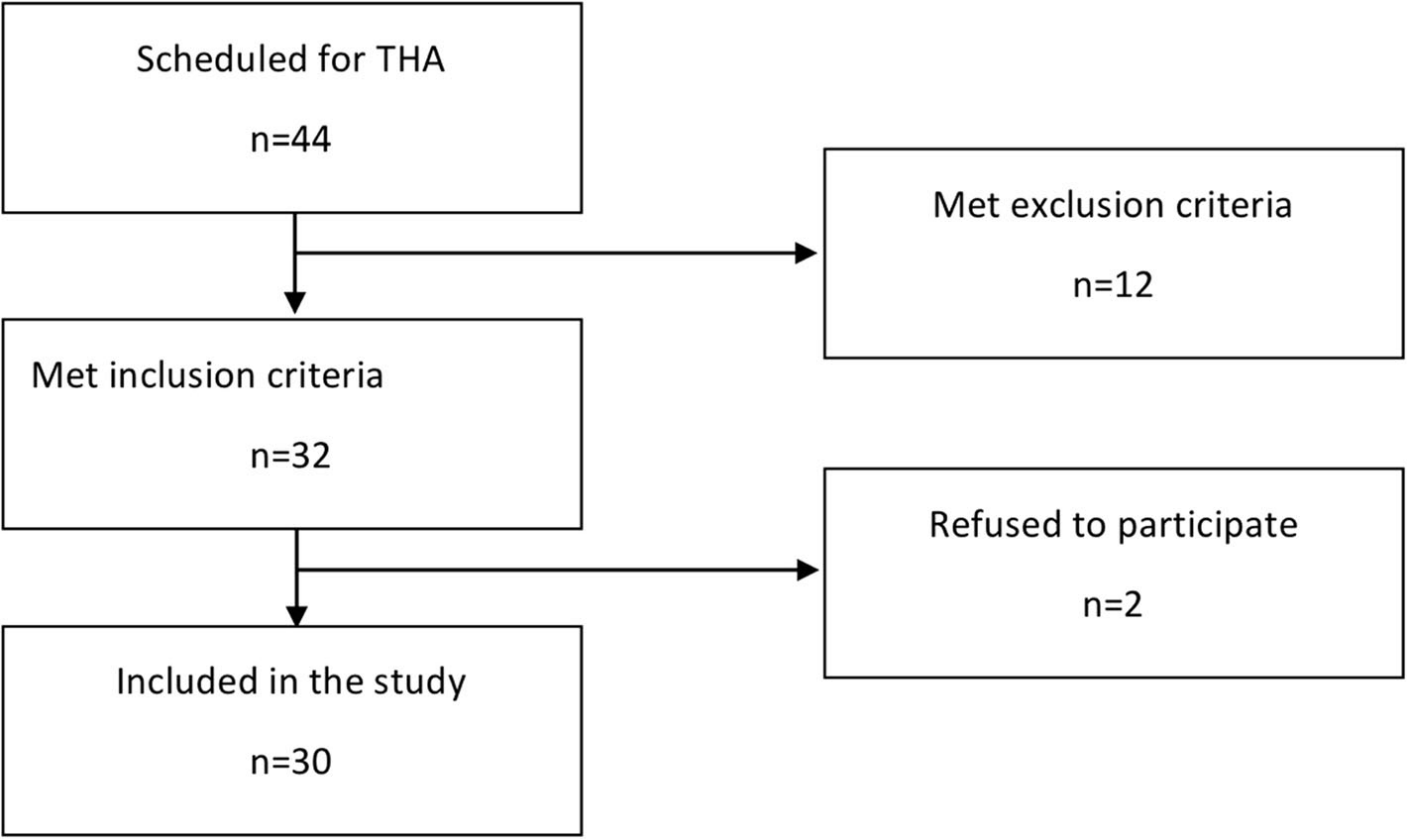
Study flow chart.jpg

Forty-four patients scheduled for hip arthroplasty were hospitalized, and all of them qualified for hip replacement; however, only 32 met the inclusion criteria, and two refused to participate in the study. Finally, data from 30 patients were analyzed. (Fig.1.)

In the study group, 18 patients were women, and the average age was 67 years (63–72.2 years). Average BMI was 26 kg/m^2^ (21–29 kg/m^2^), and an average ASA score was II (range, I-III). Preoperative hemoglobin level was 13.1 g/dL (12.9–15.4 g/dL) and postoperative hemoglobin level on the 2nd day was 10.5 g/dL (9.9–11.1 g/dL). Transfusions were not needed, and VTE symptoms were not observed during patients’ hospital stay and up to 6 weeks postoperatively. Mean blood loss was 1332 mL (range, 183–2479 mL).

**Fig. 2:**
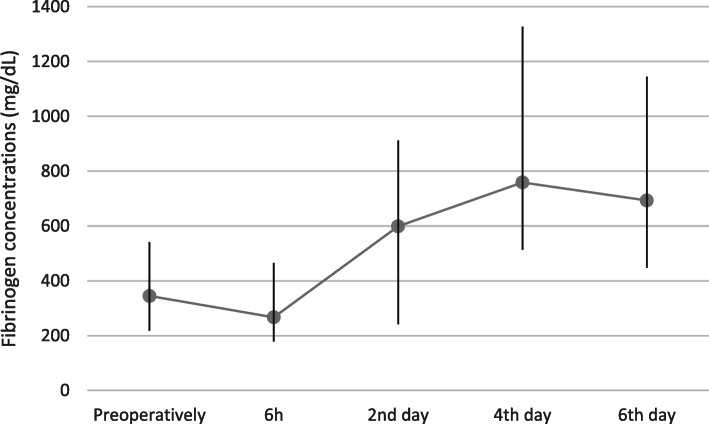
Fibrinogen concentrations (mg/dL) before and, 6 h, 2,4, and 6 days after total hip replacement surgery (Average; Min, Max)

Changes in fibrinogen concentration are shown in Fig. 2. Increased initial fibrinogen values (above the reference level of 400 mg/dL) were observed in 6 out of 30 (20%) patients. Average fibrinogen concentrations were higher than the initial levels 6 hours after the procedure and on the 2nd, fourth and sixth days post-surgery (all with *p* < 0.0001). For all patients, fibrinogen concentrations exceeded the reference level on the 4th and sixth days post-THA.

**Fig. 3:**
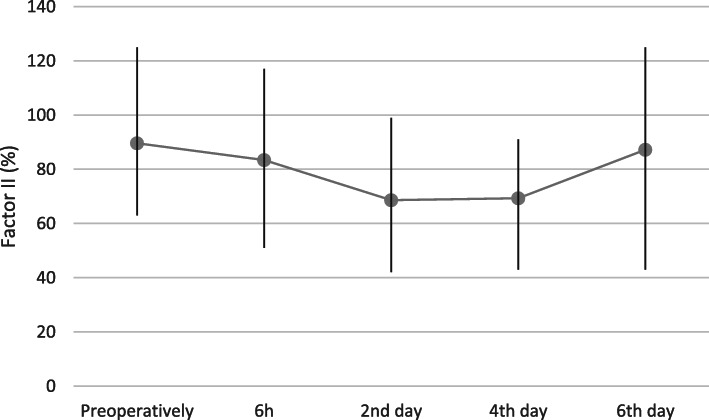
Factor II activity (%) before, and at 6 h and, 2,4 and 6 days after total hip replacement surgery (Average; Min, Max)

**Fig. 4:**
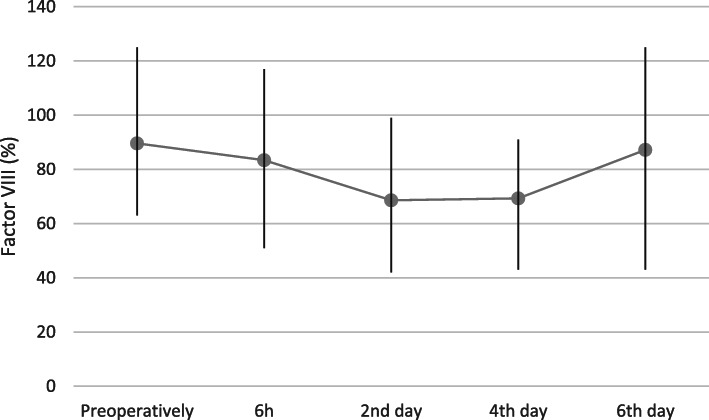
Factor VIII activity (%) before and 6 h, 2,4 and 6 days after total hip replacement surgery (Average; Min, Max)

**Fig. 5:**
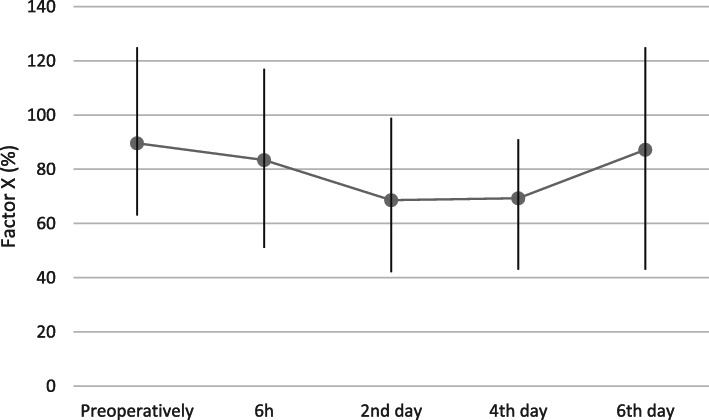
Factor X activity (%) before and 6 h, 2,4 and 6 days after total hip replacement surgery (Average; Min, Max)

Factor II activity decreased significantly (*p* < 0.0001) on the 2nd and fourth days post-surgery. Average factor VIII activity was significantly (*p* < 0.0001) lower between 2nd and sixth-day.

post-surgery. Significantly reduced factor X activity was recorded from 6 h (*p* = 0.009) until the 4th day after the procedure (*p* < 0.0001). Average clotting factor activity values with 95% confidence intervals and value ranges are presented in Figs. 2, 3, 4 and 5.

None of the demographic and laboratory parameters before the operation or immediately during the first two postoperative days could predict blood loss in a multiple regression model.

## Discussion

This study’s most important finding is that fibrinogen acts as an acute-phase protein in patients who underwent THA. Activities of clotting factors II and X normalizes within 6 days, and although the activity of factor VIII decreases, it remains within a normal range; however, changes in clotting factor activities could not predict the extent of blood loss.

A decrease in fibrinogen concentration was recorded on the day of the procedure, probably because of blood loss and increased coagulation processes immediately after the surgery. All patients had significantly elevated fibrinogen concentrations from the second to the sixth-day post THA, concordant with previous research findings [24, 34]. Elevated fibrinogen concentrations found throughout the study period are similar to the results obtained by Olsner et al., who analyzed the acute-phase response in patients undergoing THA [21].

Elevated fibrinogen concentrations were found in six out of 30 patients (20%) before THA, which is likely a manifestation of hip osteoarthritis inflammatory component or the influence of comorbidities [29]. Although increased fibrinogen concentration remains one of three Virchow’s triad components, no cases of VTE were observed in the study group. This finding also remains concordant with other papers [24] and is probably because of coexisting intensified fibrinolysis occurring in parallel with clotting system activation [2].

Surgical trauma is associated with increased systemic activities of procoagulant and anticoagulant pathways of coagulation [8]. In this study, clotting factors II, VIII, and X fluctuated; however, their activities never exceeded reference values. A slight decrease in factor II activity on the second-day post-surgery was recorded, probably because the intraoperative and postoperative bleeding was not enough to deplete it [7]. Houghton et al. reported 19-fold higher concentrations of clotting factor II in the operated limb than in the rest of the circulation, believed to result from local accumulation [12]. In the perioperative period, a slow reduction in the activity of factor VIII was noted. The opposite situation could have been expected, as factor VIII reacts to the inflammatory processes (and healing) with increased activity [1]. Its increased activity is associated with the production of interleukin 6. Interleukin 6 acts as a prothrombotic agent, increases fibrinogen concentration, activities of factors VIII and von Willebrand Factor, activates endothelial cells and platelet production and reduces levels of hemostatic inhibitors, such as antithrombin and protein S [14]*.* Perhaps low molecular weight heparin may have caused a drop in the said factor activity because heparin, apart from its anticoagulant activity, displays an anti-inflammatory effect [36]. A slight decrease in factor X activity was observed on the second-day post-surgery with a parallel change in factor II activity; it was most probably caused by low molecular weight heparin administration [30]*.*

Our study had some limitations. This study consisted of a relatively small group of patients and did not evaluate factors potentially influencing the concentration levels of coagulation factors. However, all patients referred for THA in this study were free from infection foci, their comorbidities were well managed, and the perioperative risk levels were limited (ASA I - III). In this study, blood samples were collected preoperatively and after 2, 4, and 6 days, which might have caused us to miss the actual peak and nadir times. There is some variability in methodologies used in this aspect—another research group studied clotting factor activities alone pre-and postoperative, [24] and other groups studied the same at four weeks only after surgery [32]. Another limitation is the fact that this was a single-center study.

After THA, marked blood loss may lead to higher transfusion rates, which may negatively affect surgical outcomes and lead to higher complication rates [9]. It would, therefore, be ideal to identify factors that may increase the likelihood of blood loss. Neither demographic factors nor changes in clotting factor activities or concentrations predicted blood loss in this study. Because this study was of a pilot nature, we can foresee at least three ways to improve our understanding of blood loss mechanisms after THA and identify patients at risk of significant blood loss. First, analyzing other clotting factor concentrations could complement the results presented herein. Second, to reduce blood loss after THA, TXA, an anti-fibrinolytic agent, is widely used [6]. In selective patients, administration of TXA reduced the need for blood transfusions by one third [11] and significantly reduced blood loss [6]. Therefore, it might be interesting to study clotting factor concentrations in patients being administered TXA. Third, analyzing clotting factor activities might not be an optimal method to assess the coagulation-fibrinolysis balance. Future research should focus on more responsive methods of monitoring the coagulation cascade, especially in patients at risk of significant postoperative bleeding. One of the promising methods is thromboelastography. It is performed on a blood sample to assess clot formation’s viscoelastic property under low shear conditions [22]. Thromboelastography has been successfully used to manage patients in trauma, perioperative care, and hemophilic patients [10].

Fibrinogen behavior is similar to that of an acute-phase protein—its concentration drops temporarily on the day of surgery (6 h after surgery) and increases after that. Elevated fibrinogen concentration persisted from days 2 to 6 post THA, with a maximum concentration recorded 4 days after the procedure. These changes in concentrations are probably associated with the inflammation that occurs during the healing process. No significant decrease in blood clotting factors was observed in patients undergoing THA. The use of low-molecular-weight heparin may be responsible for a slight decrease in activities of factors VIII and X. Blood loss was not correlated with changes in clotting factor concentrations and changes in clotting factors’ activities.

## Data Availability

All data generated or analysed during this study are included in this published article.
